# A randomised feasibility trial comparing needle fasciotomy with limited fasciectomy treatment for Dupuytren’s contractures

**DOI:** 10.1186/s40814-019-0546-y

**Published:** 2020-01-30

**Authors:** T. R. C. Davis, W. Tan, E. F. Harrison, W. Hollingworth, A. Karantana, N. Mills, T. Hepburn, K. Sprange, L. Duley, J. M. Blazeby, C. G. Bainbridge, S. R. Murali, A. A. Montgomery, the HAND-1 Study group

**Affiliations:** 1 Nottingham University Hospitals NHS Trust, Queen’s Medical Centre, Derby Road, Nottingham, NG7 2UH UK; 2 Nottingham Clinical Trials Unit, University of Nottingham, Building 42, University Park, Nottingham, NG7 2RD UK; 3 Bristol Medical School, University of Bristol, Canynge Hall, 39 Whatley Road, Bristol, BS8 2PS UK; 4 Centre for Evidence Based Hand Surgery, Academic Orthopaedics Trauma and Sports Medicine, School of Medicine, University of Nottingham, Nottingham, NG7 2UH UK; 5 Derby Teaching Hospitals NHS Foundation Trust, Uttoxeter Road, Derby, DE22 3NE UK; 6 Wrightington, Wigan and Leigh NHS Foundation Trust, Hall Lane, Appley Bridge, Wigan, Lancashire WN6 9EP UK

**Keywords:** Dupuytren’s contracture, Needle fasciotomy, Limited fasciectomy, Randomised trial, Feasibility

## Abstract

**Purpose:**

The purpose of this study is to assess the feasibility of conducting a large, multicentre randomised controlled trial (RCT) comparing needle fasciotomy with limited fasciectomy for treatment of Dupuytren’s contractures.

**Design:**

The design of this study is a parallel, two-arm, multicentre, randomised feasibility trial with embedded QuinteT Recruitment Intervention.

**Participants:**

Patients aged 18 years or over who were referred from primary to secondary care for treatment of a hand with Dupuytren’s contractures of one or more fingers of more than 30° at the metacarpophalangeal (MCP) and/or proximal interphalangeal (PIP) joints and well-defined cord(s). Patients were excluded if they had undergone previous Dupuytren’s contracture surgery on the same hand.

**Methods:**

Potential participants were screened for eligibility. Recruited participants randomised (1:1) to treatment with either needle fasciotomy or limited fasciectomy and followed-up for up to 6 months after treatment. Data on recruitment rates, completion of follow-up, and procedure costs were collected. Four patient reported outcome measures (PROMs) and objective outcome measures were collected before intervention and 6 weeks and 6 months afterwards.

**Results:**

One hundred and fifty-three of 267 (57%) primary-care referrals for Dupuytren’s contractures met the eligibility criteria for the study. Seventy-one of the 153 (46%) agreed to participate and were randomly allocated to treatment with needle fasciotomy or limited fasciectomy. Sixty-seven of these underwent their allocated treatment, two were crossovers from limited fasciectomy to needle fasciotomy, and two (both allocated limited fasciectomy) received no treatment. Fifty-nine participants (85%) completed 6-month follow-up PROMs. Participants felt the MYMOP, PEM and URAM PROMs allowed them to better describe how their treatment affected their hand function than the DASH PROM. The estimated costs of limited fasciectomy (in an operating theatre) and needle fasciotomy (in a clinic room) were £777 and £111 respectively.

**Conclusion:**

A large RCT comparing treatment of Dupuytren’s contractures by needle fasciotomy and limited fasciectomy is feasible. Data from this study will help determine the number of sites and duration of recruitment required to complete an adequately powered RCT and will assist the selection of PROMs in future studies on the treatment of Dupuytren’s contractures. (Level 1 feasibility study).

**Trial registration:**

Trial registered with ISRCTN (registration number: ISRCTN11164292), date assigned - 28/08/2015.

## Introduction

Needle fasciotomy (NF) and limited fasciectomy (LF) are established surgical treatments offered to patients with Dupuytren’s contractures of the fingers. NF can be undertaken in a clinic room and has a short recovery period, whereas LF is performed in an operating theatre and has a longer recovery period. A systematic review of surgery for Dupuytren’s contracture [1] found one small randomised controlled trial (RCT) comparing these treatments, which showed that LF corrects contractures better [2] and has a lower recurrence contracture rate [3] than NF. However, NF is cheaper than LF, carries a lower risk of complications and can be successfully repeated [4]. The systematic review also highlighted that the primary outcome of most existing studies is based on angular deformity of the finger joints. Though popular with health professionals, this is not a good surrogate for hand function as it does not consider the impact of treatment complications, such as finger numbness, loss of flexion and pain, on hand function [1]. Patient-centred studies, which assess function using appropriate, relevant Patient-Reported Outcome Measures (PROMs), are required to guide day-to-day clinical practice.

It is not known which of NF and LF preserves hand function better and is more cost-effective in the long term, and a large, multicentre RCT is needed to address these uncertainties. However, there is insufficient information available to effectively plan a multi-centre RCT of LF versus NF. A feasibility study which “*asks whether something can be done*, *should we proceed with it, and if so, how?*” is therefore needed [5]. The overall aim of the present study was to investigate the feasibility of conducting an RCT comparing NF with LF. This paper presents data regarding patient eligibility, recruitment and retention rates, and the performance of potential primary and secondary endpoints for clinical and cost-effectiveness. A QuinteT Recruitment Intervention (QRI) [6] to identify difficulties and optimise the recruitment process was embedded within the study and qualitative interviews were undertaken to explore patient experience. These are reported separately (Husbands S, Elliott D, Davis T, Blazeby J, Harrison E, Montgomery A, et al.: Optimising recruitment to the HAND-1 RCT feasibility study: integration of the QuinteT Recruitment Intervention (QRI), submitted to Pilot Feasibility Stud 2019).

## Methods

The trial was registered prior to the start of recruitment (ISRCTN11164292, 28 August 2015). The protocol provides details of the study design and methods [7]. Therefore, the methods are only briefly described here.

### Trial design, eligibility criteria, setting and location

This was a parallel, two-arm, three centre, randomised controlled feasibility trial. The inclusion criteria were aged 18 years or over; one or more fingers with a DC of more than 30° in the metacarpophalangeal (MCP) and/or proximal interphalangeal (PIP) joints; well-defined cord(s) causing contracture; no previous DC surgery on the same hand. The exclusion criteria were DC of the distal interphalangeal (DIP) joint only; planned dermofasciectomy or very limited fasciectomy (excision of a 1 cm or smaller cord segment); previous recruitment into this study; life expectancy less than 3 years. Recruitment took place in Hand Surgery clinics in three secondary care sites in England: Derby Teaching Hospitals NHS Foundation Trust, Wrightington, Wigan and Leigh NHS Foundation Trust and Nottingham University Hospitals NHS Trust/Nottingham Treatment Centre.

### Participant screening, recruitment and consent

Between November 2015 and September 2016, NHS patients referred to Hand Clinics at the three sites were assessed for eligibility to the trial. At one hospital, referral letters were screened by the site principal investigator to direct patients who appeared to satisfy the eligibility criteria to “recruiting clinics” where they could be invited to participate in the study. In the second hospital, referral letters were not screened and potential participants were not guided to recruiting clinics. In the third, all GP referrals with Dupuytren’s contractures were invited to opt into this study by volunteering to attend a research clinic.

Before their clinic appointment, patients were sent an information leaflet about the study. At the appointment, the treating clinician assessed patients for study eligibility, explained the study to eligible patients, and invited them to participate. Those who were interested in participating were seen by a researcher at the same clinic appointment, written informed consent was given, and the participant was randomised. Potential participants who wished to have more time to consider joining the study were offered another clinic appointment for consent and randomisation. The reasons why eligible patients chose not to participate were recorded.

### Interventions

Following randomisation, participants were placed on the waiting list for their allocated treatment. NF was performed in a clinic room with a hypodermic needle (21G: green or 23G: blue) using minimal local anaesthetic. LF was performed in an operating theatre under general or regional anaesthetic, using the surgeon’s favoured skin incision. For contractures involving the MCP joint, the cord was excised proximally to at least the proximal margin of the transverse fibres of the palmar aponeurosis. Digital cords were excised completely from their origin. In all cases, the distal margin of the cord excision was the insertion of the cord onto the flexor tendon sheath (or other structure).

### Outcomes

Feasibility outcomes were:

1. Recruitment: Number of participants assessed for eligibility, number and proportion of those assessed who were eligible, reasons for non-eligibility, number and proportion of patients randomised;
2. Retention: number and proportion of randomised participants attending 6 week and 6-month post-treatment follow-up visits and reasons for non-attendance;
3. Identification of primary and secondary outcomes for a multicentre RCT.

Four Patient-Reported Outcome Measures (PROMs) were assessed as potential primary outcome measures for a definitive RCT. These were the Unité Rhumatologique des Affections de la Main (URAM) [8], Disabilities of the Arm, Shoulder and Hand Questionnaire (DASH) [9]; Part 2 (10 questions) of the Patient Evaluation Measure (PEM) [10] and the Measure Yourself Medical Outcome Profile (MYMOP) [11, 12]. The MYMOP is a generic, patient-generated (individualised) outcome questionnaire which is brief and simple to administer. It is symptom and activity-specific but includes general wellbeing [13, 14]. Participants completed the four PROMS at the recruitment clinic before randomisation, and at 2 weeks, 6 weeks and 6 months post-treatment. The PEM was also completed on the day of treatment to monitor for change in status between recruitment and treatment, whilst on the NHS waiting list. The PROMs were assessed to determine their appropriateness for use as the primary outcome measure in a definitive RCT by: (a) recording completion rates at each time point; (b) asking each participant at each time point to record for each PROM how relevant it was to how their hand looked, felt and worked at that time on a five-point Likert scale (strongly agree, agree, neither agree nor disagree, disagree, strongly disagree) and; (c) calculation of the minimally clinical important difference (MCID).

Other outcomes collected included:

- Global Rating of Change Scale (GRC) at 6 months [15]. This acted as the anchor for the assessment of the performance of the PROMs and calculation of their MCIDs;
- Angular measurement of MCP and PIP contractures (hand-held goniometer) at baseline, 6 weeks and 6 months;
- Complications of treatment (i.e. nerve or tendon injury, finger stiffness, complex regional pain syndrome);
- Adverse events. This was limited to three serious adverse events: death due to any cause, loss of finger due to any cause and any unexpected serious events potentially related to the intervention.
- Health-related quality of life as measured by the generic EQ-5D-5 L questionnaire [16];
- Return to work and NHS resource use;
- A micro-costing of the two procedures.

The only change made to the trial assessments or outcomes during the conduct of the trial was to bring forward the 6-month questionnaire completion of participants recruited within the final 6 months before the study closed.

### Sample size

As this was a feasibility study, a sample size calculation based on power to detect a pre-specified between-group difference in a primary outcome was not appropriate. We aimed for a target sample size of 50–85 based on the assumption that three centres would assess 600 patients with Dupuytren’s contractures during the recruitment period, of whom 400 (67%) would be eligible and invited to participate, and that 13–21% of invited patients would consent and be randomised. This would enable estimation of recruitment fraction for a future study to within 5 percentage points (margin of error = half-width of 95% confidence interval), and within 14 percentage points for binary outcomes assessed among randomised participants.

### Randomisation

Participants were randomised (1:1) to treatment with either NF or LF using a secure internet-based system. This was maintained by the Nottingham Clinical Trials Unit (NCTU) in accordance with their standard operating procedures. No one directly involved in the study had access to the allocation codes. Randomisation was stratified by the recruiting centre and by the joints affected (MCP only, PIP only or MCP+PIP), and used computer-generated, permuted balanced blocks of randomly varying size. Blinding of the surgeons, researchers performing the clinical assessments and participants to intervention allocation was not possible.

### Statistical methods

We used appropriate descriptive statistics to describe recruitment data, baseline characteristics of participants, compliance with allocated intervention, completeness of data collection, and outcomes at follow-up. The statistical analysis plan was finalised before database lock and release of treatment codes to the statistician.

Participants’ opinions of the PROMs were used to guide the choice of which to use as the primary outcome in a future definitive trial. We planned to estimate the MCID and responsiveness using effect size for each of the four PROMs using the GRC as the “anchor” [17]. However, neither was possible because an insufficient number of participants (*n* = 7) reported feeling a “little better” on the GRC.

Formal between-group comparison of outcomes is not appropriate in a feasibility study [18]. We, therefore, present data by treatment group using only descriptive statistics.

### Costing methods

We used a combination of information collected on the trial case report form and clinical opinion to provide cost data for the two procedures. The case report form included details of the duration of each procedure (from introduction of anaesthetic until the patient left the procedure room), anaesthetic type (general, regional, local), surgeon(s) (consultant and/or trainee), splint provision and length of stay (admission date until discharge date). We used clinical opinion to estimate the other operating room staff, the consumables, and reusable equipment required for the NF and LF procedures. The unit cost (in 2017) of each resource was obtained from the hospital finance departments at either the Nottingham University Hospitals or the Nottingham Treatment Centre. Staff costs were estimated using NHS ‘agenda for change’ pay rates or, for consultants, per session rates. For both procedures, we included overhead costs at 30% to account for shared hospital costs (e.g. administration and utilities).

## Results

### Recruitment

**Fig. 1:**
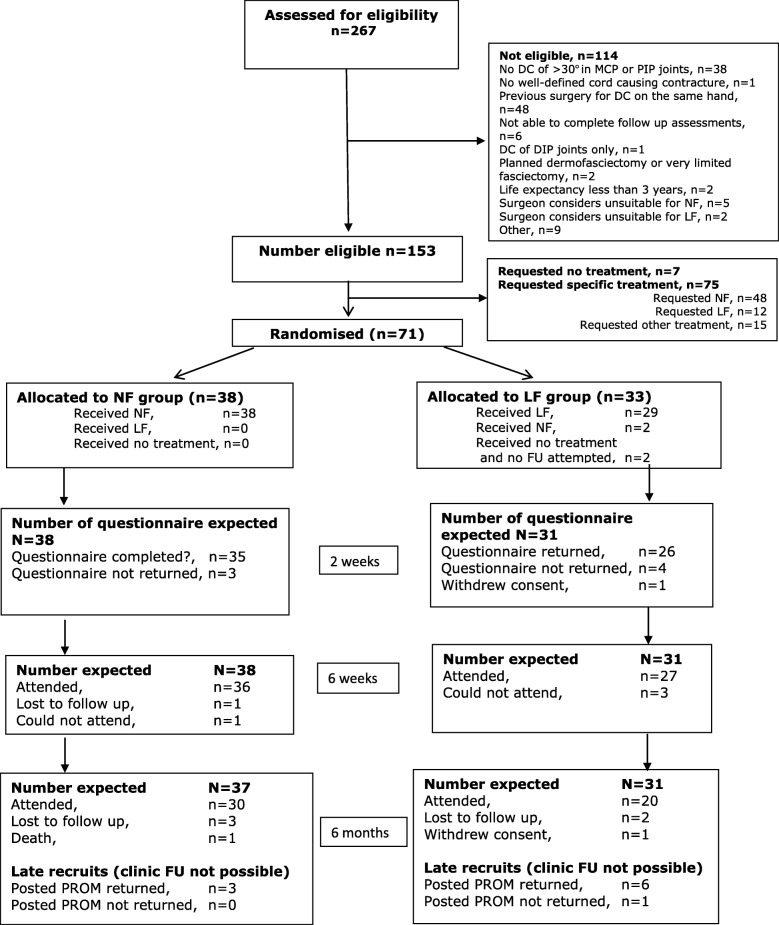
Flowchart reporting numbers of patients assessed for eligibility and recruited and randomised, and numbers of participants followed up at each timepoint

Of 267 primary care referrals with a primary (not a recurrent) Dupuytren’s contracture who were screened, 153 (57%) were eligible (Fig. 1). The most common reasons for ineligibility were previous Dupuytren’s surgery to another finger of the same hand and a contracture of 30° or less. Other exclusion criteria were infrequent and “life expectancy less than 3 years” was difficult to determine. Only seven cases were excluded as the surgeon considered them unsuitable for treatment by either NF or LF. Seventy-one of the 153 (46%) eligible patients consented to be randomised to treatment with NF or LF. Of the 82 patients who were eligible but not randomised, 75 preferred a specific treatment, of which 48 opted for NF. The remaining seven opted for no treatment (Fig. 1). Baseline demographic characteristics and PROM data were balanced between the two treatment arms (Table 1).

**Table 1:** Demographic and clinical characteristics at baseline

	Needle fasciotomy (*n* = 38)	Limited fasciectomy (*n* = 33)
Age (years)		
Mean[SD]	66.9 [7.1]	64.4 [7.8]
Gender		
Male	27 (71%)	27 (82%)
Female	11 (29%)	6 (18%)
Ethnicity		
White	38 (100%)	33 (100%)
Right or left handed		
Right	30 (79%)	27 (82%)
Left	8 (21%)	5 (15%)
Missing	0 (0%)	1 (3%)
Study hand		
Right	19 (50%)	22 (67%)
Left	19 (50%)	11 (33%)
Dominant hand affected	17 (45%)	18 (54%)
Study finger		
Index	0	0
Little	20 (53%)	21 (64%)
Middle	5 (13%)	4 (12%)
Ring	13 (34%)	8 (24%)
Joints affected on study finger		
MCP joint only	12 (32%)	10 (30%)
PIP joint only	12 (32%)	10 (30%)
MCP and PIP joints	14 (37%)	13 (39%)
Grip strength for trial hand (kgf), mean[SD]	28.2 [12.3]	30 [11.1]
Grip strength for non-trial hand (kgf), mean[SD]	30.5 [10.9]	32.6 [10]
Extension angular measurement (degrees), mean [SD]		
MCP joint	43.4 [19]	47.3 [19.9]
PIP joint	45.4 [17.1]	44.8 [20.4]
DIP joint	25.4 [25.3]	34.3 [18.2]
DASH score, mean[SD]	20.3 [19.4]	20.9 [15.3]
PEM score, mean[SD]	27.2 [13.8]	29.8 [12.3]
URAM score, mean[SD]	19.5 [11]	21.3 [11.6]
MYMOP profile score, mean[SD]	3.1 [1.1]	3.3 [1.3]
EQ-5D-5 L score, mean[SD]	0.808 [0.200]	0.813 [0.175]

All data are *N* (%)’s unless specified

*SD* Standard deviation

Patients with involvement of MCP joint only, PIP joint only and both MCP and PIP joints were recruited at all three centres, showing willingness of surgeons to recruit patients with each of these different DC patterns (Table 1).

### Compliance with allocated treatment

Sixty-seven patients received their allocated treatment. Two patients randomised to LF received NF due to preferences and another two did not receive surgery before the study closed (Fig. 1).

### Retention and follow-up

There were delays between randomisation and treatment due to NHS waiting lists for surgery: median 97 days for LF and 41 days for NF. As follow-up was timed from the treatment rather than randomisation, this resulted in 10 of the 69 participants who received their treatment not attending a 6-month clinic follow-up by the end of the study. Nine of these participants returned questionnaires sent by post earlier than 6 months (the earliest was 20 weeks after treatment).

Sixty-three (91%) of the 69 treated participants attended their 6-week follow-up appointment and 50 (85%) of the 59 who could have attended their 6-month follow-up before the study closed did so. On average, the timing of follow-up closely matched the planned schedule at 2 and 6 weeks and 6 months (Additional file 1: Table S1).

### Outcome completion

Data collected on the day of surgery was over 90% complete. This detailed the duration of surgery and hospital stay, surgeon status (consultant/trainee), the precise surgery performed, recognition of any immediate surgical complications (i.e. nerve injury), and the immediate effect of the intervention on contracture.

Data on hand grip strength and angular measurement of persistent finger deformity at 6 weeks were complete in over 88% of the 69 treated participants and over 80% of the 50 participants who could have attended the 6-month post-surgery assessment clinics.

Over the whole follow-up period of the study, 753 of 887 PROMs (85%) were completed sufficiently for analysis (Additional file 2: Table S2).

On the day of surgery, the PEM was completed sufficiently for evaluation in 60 (87%) of the 69 treated participants. The 2-week post-surgery PROMS, which were posted to participants for completion at home, were returned by 61 (88%) of the 69 treated participants and each of the four individual PROMs was evaluable in 75% or more instances. At the 6-week post-surgery assessments, the PROMs were completed in clinic, and were completed sufficiently for evaluation in 85% or more instances. At 6 month follow-up, when 50 of the 59 participants completed the PROMs in the clinic and nine by post, the completion rate was again at least 85%.

### PROM assessment

Forty-six (78%) of 58 participants who completed the GRC at 6 months reported feeling “much better”, seven (12%) “a little better”, one (2%) “unchanged” and four (7%) “a little worse”. Mean scores were improved for each of the four PROMS at 6 months among participants reporting improvement on the GRC (Table 2).

**Table 2:** Change from baseline score at 6 months for each of the PROMS according to self-reported global assessment of change at 6 months post-surgery

	GRC at 6 months Worse or about the same	GRC at 6 months A little better or much better
DASH (possible range 0–100)		
Mean (SD)	6.5 [9.9]	−13.8 [16.8]
Median (25^th^, 75^th^ centile)	6.2 [−2.1, 15]	−11.7[−17.9, −3.7]
Min, max	−2.5, 15.8	−60, 42.5
*N*	4	52
PEM (possible range 0–70)		
Mean (SD)	8.8 [3.8]	−19.0[14.0]
Median (25^th^, 75^th^ centile)	8 [7, 11]	−18[−30, −11]
Min, max	4, 14	−55, −26
*N*	5	51
URAM (possible range 0–45)		
Mean (SD)	2.0 [1.4]	−16.7 [11.4]
Median (25^th^, 75^th^ centile)	1.5 [1, 3]	−16.5 [−26, −8]
Min, max	1, 4	−40,8
*N*	4	50
MYMOP (possible range 0–6)		
Mean (SD)	−0.1 [1.2]	−2.3 [1.2]
Median (25^th^, 75^th^ centile)	0.3 [−1.2, 0.6]	−2.3 [−3, −1.6]
Min, max	−1.5, 1.3	−5.7, 0
*N*	5	53

For all four PROMS, a lower score indicates a greater improvement from baseline.

*SD* Standard deviation

Table 3 shows the change from baseline for each PROM at 6 weeks and 6 months. Summary PROM data at baseline and follow-up by treatment arm are shown in Table 4.

**Table 3:** Summary of PROMs change from baseline at 6 weeks and 6 months for all participants

	Change from baseline Mean [SD] Median [25^th^, 75^th^ Centile]	
	At 6 weeks	At 6 months
DASH	−7.7 [14.8]	−12.4 [17.2]
	−7.5 [−16.4, −1.7]	−9.6 [−17.5, −2.9]
PEM	−14.8 [15.2]	−16.5 [15.6]
	−15 [−26, −8]	−17.5 [−28, −7.5]
URAM	−13.6 [12.1]	−15.3 [12.1]
	−13 [−22, −5]	−13 [−25, −7]
MYMOP profile score	−2 [1.2]	−2.1 [1.4]
	−2[−3, −1.2]	−2.2 [−3, −1.5]

*SD* standard deviation

**Table 4:** Summary of PROM scores by allocated group, at baseline and 6 months

	Baseline		6 months	
	Needle fasciotomy *N* = 38	Limited fasciectomy *n* = 33	Needle fasciotomy *N* = 33	Limited fasciectomy *n* = 26
DASH, mean[SD]	20.3 [19.4]	20.9 [15.3]	9.3 [16.2]	7.0 [7.9]
PEM, mean[SD]	27.2 [13.8]	29.8 [12.3]	10.8 [15.4]	13.3 [14.5]
URAM, mean[SD]	19.5 [11]	21.3 [11.6]	4.8 [6.7]	4.2 [6]
MYMOP profile score, mean[SD]	3.1 [1.1]	3.3 [1.3]	1.1 [1.3]	1.0 [1]
EQ5D-5 L, mean[SD]	0.808 [0.200]	0.813 [0.175]	0.915 [0.145]	0.881 [0.181]

*SD* Standard deviation

A high proportion of participants reported the PROMS as relevant to their hand condition at baseline and follow up (Additional file 3: Table S3). The DASH had lower reported relevance than the other PROMS at baseline and 2 weeks.

The mean PEM score increased slightly in both the NF (2.9: SD = 11.5, *N* = 33) and LF (3.3: SD = 7.1, *N* = 26) groups between baseline and the day of treatment, whilst waiting for treatment.

Correlations between changes from baseline to 6 months in the four PROM scores and changes in angular measurement (using a goniometer) of the treated finger were weak (DASH 0.387, PEM 0.382) to moderate (MYMop 0.598, URAM 0.507).

### Correction of finger deformity

Fifty-two of the 60 MCP joint contractures were fully corrected by treatment (LF or NF). Twenty-two of 47 contractures of the PIP joint were fully corrected. The median residual proximal interphalangeal joint contracture was 20°. Summary data for correction of deformity and grip strength at baseline and follow-up by treatment arm are shown in Table 5.

**Table 5:** Summary of clinical outcomes by allocated group, at baseline and 6 months

		Baseline		6 months	
		Needle fasciotomy *N* = 38	Limited fasciectomy *n* = 33	Needle fasciotomy *N* = 30	Limited fasciectomy *n* = 20
Grip strength for trial hand (kgf)					
	Mean[SD]	28.2 [12.3]	30 [11.1]	30.4 [13.1]	28 [9.4]
Grip strength for non-trial hand (kgf)					
	Mean[SD]	30.5 [10.9]	32.6 [10]	30.9 [11.2]	37.6 [8.6]
Extension angular measurement (degrees)					
MCP joint	Mean[SD]	43.4 [19]	47.3 [19.9]	3.8 [5.9]	2.4 [5.4]
PIP joint	Mean[SD]	45.4 [17.1]	44.8 [20.4]	20.3 [19.8]	15.5 [19.2]
DIP joint	Mean[SD]	25.4 [25.3]	34.3 [18.2]	3.1 [8]	4.8 [12.4]

*SD* Standard deviation

### Complications of surgery

These are summarised in Table 6. Complications were uncommon, but not negligible and a larger study would be needed to compare between the treatments.

**Table 6:** Summary of complications following treatment

	NF group (*N* = 38)	LF group (*N* = 33)
6 weeks post-surgery		
Delayed wound healing	0	2
Numbness	1	2
Loss of flexion in operated finger	1	1
Loss of flexion in one or more fingers	2	3
Complete division of flexor tendon	0	0
Complex regional pain syndrome	0	0
Other complication	1	1
6 months post-surgery		
Delayed wound healing	0	0
Numbness	3	0
Loss of flexion in operated finger	2	4
Loss of flexion in one or more fingers	2	3
Complete division of flexor tendon	0	0
Complex regional pain syndrome	0	0
Loss of finger	0	0
Other complication	4	1

Data are number of participants with complications. Numbness was not quantified and does not necessarily indicate a digital nerve injury

### Serious adverse events

There was only one serious adverse event which was the unrelated death of one participant. No safety concerns were identified.

### Costs and resource use

The mean (SD) procedure times were 88.2 (23) and 19.4 (9) minutes for LF and NF respectively, a difference of 68.7 minutes (95% CI 60.3 to 77.1). Most (72%) of LF procedures were performed using a regional block anaesthetic. Most patients (83%) who had LF were prescribed a finger splint compared to the minority of patients (32%) who had NF. The estimated total cost of the LF procedure, excluding the costs of a pre-operative assessment, finger splints and time spent in the recovery room and on the day case ward, was £777 (Table 7). One patient who had LF required a two-night stay in hospital which would have incurred additional costs. The estimated total cost of the NF procedure was £111 (Table 8).

**Table 7:** Micro-costing of LF procedure

Limited fasciectomy (88-minute procedure)	
Resource	£
Staffing costs	
Consultant anaesthesiologist	£192.50
Operating Department Assistant	£24.13
Theatre (scrub) nurses (×2)	£45.46
Theatre (runner) nurse	£26.82
Theatre auxiliary	£14.58
Consultant surgeon	£192.50
Registrar (in 45% of cases)	£19.56
Sub-total	£515.55
Consumable costs	
Tourniquet (disposable cuff)	£9.90
Eschmark exsanguination bandage	£1.68
Disposable light handles	£0.42
Scalpel blades	£2.32
4-0 vicryl rapide suture (2 packets)	£3.80
Gelanite	£0.37
Melanite	£0.33
3-inch plaster wool	£3.54
50 cm 3-inch plaster	£1.94
Elastoplast 15 cm	£1.09
Bradford sling	£14.53
Reusable equipment*	
Lead hand and 5 elastic bands (reusable)	£4.43
Black handled scissors	£4.43
Orthopaedic tray	£33.24
Sub-total	£82.02
Overhead cost (@30%)	£179.27
Total cost	£776.85

* As these items are re-used frequently we assumed that the capital cost per patient was negligible. These costs reflect the washing and sterilisation costs between procedures

**Table 8:** Micro-costing of NF procedure

Needle fasciotomy (18-minute procedure)	
Resource	£
Staffing costs	
Procedure room nurse—band 5	£4.65
Consultant surgeon	£39.38
Registrar (in 45% of cases)	£4.00
Sub-total	£48.02
Consumable costs	
2-ml syringe	£0.63
23-gauge (blue)hypodermic needle	£0.56
25-gauge (orange) hypodermic needle	£0.56
21-gauge (green) hypodermic needle	£0.56
Dressing pack	£0.31
Sterile drapes	£0.32
Dressings	£3.90
Alcoholic chlorhexidine	£14.10
5 ml 1% lidocaine	£16.10
Non-sterile clinic room blue gloves	£0.06
Sub-total	£37.10
Overhead cost (@30%)	£25.54
Total cost	£110.66

One patient had a limited fasciectomy in the 6 months following the initial needle fasciotomy. This patient also visited the ED with wound dehiscence approximately 10 days after the limited fasciectomy. A further two patients had a carpal tunnel release in the 6 months following the initial procedure. About one third of patients were working at all time points after the procedure (Table 9). In those who were working, mean time lost from work in the last 7 days due to hand/finger problems decreased after the procedure; no time was lost from work in the last 7 days at 6 months post-procedure.

**Table 9:** Employment status and hours lost from work in the last 7 days, complete case analysis (*n* = 46)

Timepoint	Currently working for pay (%)	Mean (SD) hours lost, among workers
2 Weeks	16/46 (35)	10.75 (16.3)
6 Weeks	17/46 (37)	4.35 (10.3)
6 Months	15/46 (33)	0 (0)

## Discussion

This study has confirmed important aspects of feasibility required for the design and conduct of a future randomised trial to compare needle fasciotomy with limited fasciectomy for treating Dupuytren’s contractures. We successfully identified, screened, invited and randomised eligible patients from three sites, and about half of eligible patients agreed to participate. Most participants received their treatment as allocated and completed clinic visits or questionnaires at up to 6-month follow-up. We found that patient-reported outcome measures were responsive to change but were not particularly strongly associated with improvements in the extension of treated finger joints, an outcome that is often used clinically to measure treatment success. A definitive trial in patients with Dupuytren’s contracture assessing the clinical and cost-effectiveness of NF and LF in terms of patient-reported outcomes is therefore feasible and acceptable in the UK and recommended to inform clinical decision-making and health policy.

We recognise that 6-month follow-up is insufficient to investigate recurrence rates following treatment, which are known to increase with length of follow-up and differ markedly for NF and LF [3]. Recurrence affects hand function, the need for further treatment and cost, and follow-up of at least 5 years would be desirable for a definitive trial comparing these two treatments.

Our feasibility findings relate to three sites, and may not be easily generalisable to all centres where Dupuytren’s contractures are treated. This is because patient pathways, length of waiting lists and the enthusiasm of surgeons to randomise patients to either NF or LF will vary from centre to centre. Some centres may be reluctant to recruit patients with contractures of the proximal interphalangeal joint as they do not consider NF appropriate in this situation. This is because of concerns it may cause nerve damage, even though the reported rate of this complication is low [19, 20] and a systematic review reports greater rates of nerve injury after limited fasciectomy [21]. Also, the stipulation to perform NF in a clinic room setting, rather than an operating theatre, may be resisted by some centres due to the idiosyncrasies of NHS reimbursement, in which £1085 is paid for a NF in an operating theatre, and only £73 for one in a clinic room. For these reasons, an embedded QuinteT Recruitment Intervention, as used in this trial, is advised within the definitive trial to identify and address site-specific barriers to recruitment in each recruiting site (Husbands S, Elliott D, Davis T, Blazeby J, Harrison E, Montgomery A, et al.: Optimising recruitment to the HAND-1 RCT feasibility study: integration of the QuinteT Recruitment Intervention (QRI), submitted to Pilot Feasibility Stud 2019).

Three of the four PROMS (MYMOP, PEM and URAM) were consistently considered relevant by participants. All could be used as the potential primary outcome for the definitive multicentre RCT. The MYMOP is not used widely, if at all, in hand surgery, whereas the PEM and URAM are widely used. The URAM was designed specifically for Dupuytren’s surgery and considers losses in function expected in association with the inability to fully straighten the fingers and does not assess pain or strength. The PEM is not disease-specific, assesses hand function more generally, and includes assessments of pain and strength. It is also the primary outcome in an ongoing multicentre study comparing the treatment of Dupuytren’s contractures by limited fasciectomy and collagenase injection (goo.gl/zhvyq8).

Despite participants in the LF group having a longer wait between randomisation and treatment than those allocated to NF, reflecting usual NHS care, we found similar mean changes in PEM scores during this period in both groups. This suggests follow-up from the day of treatment, rather than baseline, is acceptable for studies of Dupuytren’s contracture treatment, and that the longer delay between randomisation and treatment with LF, rather than NF, would not bias the results of a RCT comparing these two treatment options.

The health economics questionnaire allowed determination of time off work for those participants who were in employment. Clinic room and operating theatre costs for NF and LF were also estimated. Not surprisingly, there is a considerable difference between the costs of these two treatments. The calculated cost of needle fasciotomy is similar to the cost calculated in another study [22].

The use of questionnaires to measure both clinical and health economic outcomes potentially allows remote follow-up of participants, without the need for research clinic attendances after standard clinical follow-up is complete. Remote collection of data beyond 3 months after treatment would reflect NHS care, as most patients have been discharged from follow-up clinics by this stage. It might also increase participant retention rate. Whilst we achieved 80% follow-up at a 6-month research clinic attendance, remote follow-up might improve retention, particularly at longer follow-up and for participants who may have difficulty attending clinics. However, an objective assessment of participants in the early follow-up phase would ensure complications are detected and quantified. A 6-week clinical follow-up appointment would be suitable, and our findings suggest that numbness needs to be quantified to distinguish significant digital nerve injuries from less significant diminished sensibility. Also, the frequency and severity of recurrent contracture formation at later follow-up times is an important secondary outcome measure. A simple and reliable method for assessing finger straightness which can be undertaken by participants in their homes is needed if this is to be assessed remotely. Potential methods include digital photography and simple goniometers.

Our data assists the planning of a future multicentre RCT comparing NF with LF. It has highlighted: (a) issues with the eligibility criteria which can be improved (i.e. changing "contracture of more than 30°” to "contracture of 30° or more” and “life expectancy of less than 3 years: to “likely to be available to complete follow-up”) and; (b) shown that recruitment would be enhanced by modification to allow recruitment of patients who had previously had treatment for a Dupuytren’s contracture of another finger in the same hand. However, their previous experiences of treatment may make them reluctant to agree to recruitment, consent and randomisation.

## Conclusions

A large RCT comparing treatment of DC by NF and LF is feasible. We would recommend the number of recruiting sites and duration of such a study is determined on the basis of: (a) about 50% of referrals from primary care being eligible; (b) about 50% of those eligible being willing to be recruited and randomised to treatment and; (c) a 6-month retention rate of about 85%. Delays in provision in treatment, particularly for LF, also need to be considered to ensure the follow-up phase of the study is sufficiently long. Participants felt the MYMOP, PEM and URAM PROMs allowed them to better describe the condition of their hand than the DASH.

## Supplementary information

**Additional file 1.** Timing of follow-up.

**Additional file 2.** Completeness of follow-up.

**Additional file 3.** Number of participants agreeing each PROM allowed accurate description of their hand’s condition.

## Data Availability

The datasets used and/or analysed during the current study are available from the corresponding author on reasonable request.

## Abbreviations

DASH: Disabilities of the Arm, Shoulder and Hand Questionnaire
DC: Dupuytren’s contracture
DIP: Distal interphalangeal joint
GRC: Global Rating of Change Scale
LF: Limited fasciectomy
MCID: Minimally important clinical difference
MCP: Metacarpophalangeal joint
MYMOP: Measure Yourself Medical Outcome Profile
NCTU: Nottingham Clinical Trials Unit
NF: Needle fasciotomy
NHS: National Health Service
PEM: Patient Evaluation Measure
PIP: Proximal interphalangeal joint
PROM: Patient-reported outcome measure
QRI: QuinteT Recruitment Intervention
RCT: Randomised controlled study
URAM: Unité Rhumatologique des Affections de la Main

## References

[1] Rodrigues JN, Becker GW, Ball C, Zhang W, Giele H, Hobby J, et al. Surgery for Dupuytren's contracture of the fingers. Cochrane Database of Systematic Reviews. 2015. 10.1002/14651858.CD010143.pub2 PMC6464957 26648251

[2] van Rijssen AL, Gerbrandy FSJ, Linden HT, Klip H, Werker PMN (2006). A Comparison of the direct outcomes of percutaneous needle fasciotomy and limited fasciectomy for dupuytren's disease: a 6-week follow-up study. J Hand Surg.

[3] van Rijssen AL, ter Linden H, Werker PM (2012). Five-year results of a randomized clinical trial on treatment in Dupuytren's disease: percutaneous needle fasciotomy versus limited fasciectomy. Plast Reconstr Surg.

[4] van Rijssen AL, Werker PM (2012). Percutaneous needle fasciotomy for recurrent Dupuytren disease. J Hand Surg Am.

[5] Eldridge SM, Lancaster GA, Campbell MJ, Thabane L, Hopewell S, Coleman CL (2016). Defining Feasibility and pilot studies in preparation for randomised controlled trials: development of a conceptual framework. PLoS One.

[6] Donovan JL, Rooshenas L, Jepson M, Elliott D, Wade J, Avery K (2016). Optimising recruitment and informed consent in randomised controlled trials: the development and implementation of the Quintet Recruitment Intervention (QRI). Trials..

[7] Harrison E, Tan W, Mills N, Karantana A, Sprange K, Duley L (2017). A feasibility study investigating the acceptability and design of a multicentre randomised controlled trial of needle fasciotomy versus limited fasciectomy for the treatment of Dupuytren's contractures of the fingers (HAND-1): study protocol for a randomised controlled trial. Trials.

[8] Beaudreuil J, Allard A, Zerkak D, Gerber RA, Cappelleri JC, Quintero N (2011). Unite Rhumatologique des Affections de la Main (URAM) scale: development and validation of a tool to assess Dupuytren's disease-specific disability. Arthritis Care Res (Hoboken).

[9] Hudak PL, Amadio PC, Bombardier C (1996). Development of an upper extremity outcome measure: the DASH (disabilities of the arm, shoulder and hand) [corrected]. The Upper Extremity Collaborative Group (UECG). Am J Ind Med.

[10] Macey AC, Burke FD, Abbott K, Barton NJ, Bradbury E, Bradley A (1995). Outcomes of hand surgery. British Society for Surgery of the Hand. J Hand Surg (Edinburgh, Scotland).

[11] Paterson C (1996). Measuring outcomes in primary care: a patient generated measure, MYMOP, compared with the SF-36 health survey. BMJ.

[12] Paterson C (2004). Seeking the patient’s perspective: a qualitative assessment of EuroQol, COOP-WONCA Charts and MYMOP2. Qual Life Res.

[13] Care CfAP. MyMop website. Available from: http://www.bris.ac.uk/primaryhealthcare/resources/mymop/. Accessed 6 May 2019.

[14] Ruta D, Garratt A (1996). MYMOP, a patient generated measure of outcomes. Reliability of such instruments needs to be proved. BMJ.

[15] Kamper SJ, Maher CG, Mackay G (2009). Global rating of change scales: a review of strengths and weaknesses and considerations for design. J Man Manip Ther.

[16] Devlin N, Shah K, Feng Y, Mulhern B, van Hout B (2018). Valuing health-related quality of life: An EQ-5D-5 L value set for England. Health Econ.

[17] Rodrigues JN, Mabvuure NT, Nikkhah D, Shariff Z, Davis TR (2015). Minimal important changes and differences in elective hand surgery. J Hand Surg Eur Vol.

[18] Eldridge SM, Chan CL, Campbell MJ, Bond CM, Hopewell S, Thabane L (2016). CONSORT 2010 statement: extension to randomised pilot and feasibility trials. BMJ.

[19] Pess GM, Pess RM, Pess RA (2012). Results of needle aponeurotomy for Dupuytren contracture in over 1,000 fingers. J Hand Surg Am.

[20] Skov ST, Bisgaard T, Sondergaard P, Lange J (2017). Injectable Collagenase Versus Percutaneous Needle Fasciotomy for Dupuytren Contracture in Proximal Interphalangeal Joints: A Randomized Controlled Trial. J Hand Surg Am.

[21] Crean SM, Gerber RA, Le Graverand MP, Boyd DM, Cappelleri JC (2011). The efficacy and safety of fasciectomy and fasciotomy for Dupuytren's contracture in European patients: a structured review of published studies. J Hand Surg Eur Vol.

[22] Stromberg J, Ibsen-Sorensen A, Friden J (2016). Comparison of treatment outcome after collagenase and needle fasciotomy for dupuytren contracture: a randomized, single-blinded, clinical trial with a 1-year follow-up. J Hand Surg Am.

