# Supplementary material for: A randomised feasibility trial comparing needle fasciotomy with limited fasciectomy treatment for Dupuytren’s contractures
Source: Pilot Feasibility Stud. 2020 Jan 30;6:7. doi: 10.1186/s40814-019-0546-y (PMC6993423; doi:10.1186/s40814-019-0546-y)
Supplement: Supplementary file 1 — Additional file 1. Timing of follow-up. [file 40814_2019_546_MOESM1_ESM.docx]

Additional file 1: Table S1. Timing of follow-up.

|  | Needle Fasciotomy | Limited Fasciectomy |
| --- | --- | --- |
| Time from surgery to 2 week questionnaire completed (weeks)  Mean[SD]  Median[25^th^, 75^th^ centile]  N | 2.7 [1.1]  2.3 [2, 2.9]  35 | 2.8 [1.5]  2 [2, 3.3]  26 |
| Time from surgery to 6 week follow up (weeks)  Mean[SD]  Median[25^th^, 75^th^ centile]  N | 6.8 [1.6]  6.2 [6, 7.5]  36 | 6.5 [1.7]  6.3 [6.1, 7.3]  27 |
| Time from surgery to 6 month follow up (weeks)  Mean[SD]  Median[25^th^, 75^th^ centile]  N | 25 [3.7]  23.8 [22, 27]  30 | 24.7 [4]  23.5 [21.8, 27.7]  20 |

*SD* – standard deviation
