# Supplementary material for: A randomised feasibility trial comparing needle fasciotomy with limited fasciectomy treatment for Dupuytren’s contractures
Source: Pilot Feasibility Stud. 2020 Jan 30;6:7. doi: 10.1186/s40814-019-0546-y (PMC6993423; doi:10.1186/s40814-019-0546-y)
Supplement: Supplementary file 2 — Additional file 2. Completeness of follow-up. [file 40814_2019_546_MOESM2_ESM.docx]

Additional file 2: Table S2. Completeness of follow-up

|  | Needle Fasciotomy | Limited Fasciotomy | Total |
| --- | --- | --- | --- |
| DASH  2 weeks  6 weeks  6 months | 34/38 (89%)  34/38 (89%)  33/37 (89%) | 25/31 (81%)  26/30 (87%)  24/30 (80%) | 59/69 (86%)  60/68 (88%)  57/67 (85%) |
| PEM  Day of Surgery  2 weeks  6 weeks  6 months | 34/38 (89%)  32/38 (84%)  32/38 (84%)  33/37 (89%) | 26/33 (79%)  22/31 (71%)  26/30 (87%)  24/30 (80%) | 60/71 (85%)  54/69 (78%)  58/68 (85%)  57/67 (85%) |
| URAM  2 weeks  6 weeks  6 months | 31/38 (82%)  34/38 (89%)  33/37 (89%) | 21/31 (68%)  26/30 (87%)  24/30 (80%) | 52/69 (75%)  60/68 (88%)  57/67 (85%) |
| MYMOP  2 weeks  6 weeks  6 months | 34/38 (89%)  34/38 (89%)  33/37 (89%) | 26/31 (84%)  27/30 (90%)  25/30 (83%) | 60/69 (87%)  61/68 (90%)  58/67 (87%) |
| EQ-5D-5 L  2 weeks  6 weeks  6 months | 32/38 (84%)  34/38 (89%)  33/37 (89%) | 26/31 (84%)  27/30 (90%)  25/30 (83%) | 58/69 (84%)  61/68 (90%)  58/67 (87%) |
| Grip in treated hand  6 weeks  6 months | 34/38 (89%)  29/34 (85%) | 26/30 (87%)  17/23 (74%) | 60/68 (88%)  46/57 (81%) |
| Grip in contralateral hand  6 weeks  6 months | 34/38 (89%)  27/34 (79%) | 24/30 (80%)  17/23 (74%) | 58/68 (85%)  44/57 (81%) |
| Angle measurement (goniometer)  6 weeks  6 months | 36/38 (95%)  29/34 (85%) | 27/30 (90%)  18/23 (78%) | 63/68 (93%)  47/57 (82%) |

Denominator = number available for follow-up at timepoint. Number available differs between PROMS and objective measures at 6 months as NHS waiting lists meant 10 participants could only complete PROMS.
