# Supplementary material for: A randomised feasibility trial comparing needle fasciotomy with limited fasciectomy treatment for Dupuytren’s contractures
Source: Pilot Feasibility Stud. 2020 Jan 30;6:7. doi: 10.1186/s40814-019-0546-y (PMC6993423; doi:10.1186/s40814-019-0546-y)
Supplement: Supplementary file 3 — Additional file 3. Number of participants agreeing each PROM allowed accurate description of their hand’s condition. [file 40814_2019_546_MOESM3_ESM.docx]

Additional file 3: Table S3. Number of participants agreeing each PROM allowed accurate description of their hand’s condition

|  | Missing data | | Needle Fasciotomy | Limited Fasciotomy | Total |
| --- | --- | --- | --- | --- | --- |
|  | NF | LF |  |  |  |
| DASH  Baseline  2 weeks  6 weeks  6 months | 1  9  6  9 | 0  9  8  14 | 25/37 (68%)  22/29 (76%)  25/32 (78%)  25/29 (86%) | 24/33 (73%)  12/24 (50%)  24/26 (92%)  17/19 (89%) | 49/70 (70%)  34/53 (64%)  49/58 (84%)  42/48 (88%) |
| PEM  Baseline  2 weeks  6 weeks  6 months | 1  7  6  8 | 0  10  6  14 | 33/37 (89%)  26/31 (84%)  28/32 (88%)  29/30 (97%) | 28/33 (85%)  18/23 (78%)  24/27 (89%)  16/19 (84%) | 61/70 (87%)  44/54 (81%)  52/59 (88%)  45/49 (92%) |
| URAM  Baseline  2 weeks  6 weeks  6 months | 1  9  6  9 | 1  11  8  14 | 31/37 (84%)  24/29 (83%)  28/32 (88%)  28/29 (97%) | 27/32 (84%)  17/22 (77%)  24/25 (96%)  17/19 (89%) | 58/69 (84%)  41/51 (80%)  52/57 (91%)  45/48 (94%) |
| MYMOP  Baseline  2 weeks  6 weeks  6 months | 1  11  9  12 | 1  11  9  16 | 33/37 (89%)  22/27 (81%)  23/29 (79%)  24/26 (92%) | 29/32 (91%)  18/22 (82%)  21/24 (88%)  13/17 (76%) | 62/69 (90%)  40/49 (82%)  44/53 (83%)  37/43 (86%) |
